# Balasubramide derivative 3C attenuates atherosclerosis in apolipoprotein E-deficient mice: role of AMPK-STAT1-STING signaling pathway

**DOI:** 10.18632/aging.202929

**Published:** 2021-04-26

**Authors:** Dongcheng Cai, Hongxia Liu, Jing Wang, Yuanlong Hou, Tao Pang, Hansen Lin, Chaoyong He

**Affiliations:** 1 State Key Laboratory of Natural Medicines, Department of Pharmacology, China Pharmaceutical University, Nanjing 210009, Jiangsu, China; 2 Jiangsu Province Key Laboratory of Drug Metabolism, State Key Laboratory of Natural Medicines, China Pharmaceutical University, Nanjing 210009, Jiangsu, China; 3 Jiangsu Key Laboratory of Drug Screening, State Key Laboratory of Natural Medicines, China Pharmaceutical University, Nanjing 210009, China; 4 College of Pharmacy, Guangdong Pharmaceutical University, Guangzhou 510006, China

**Keywords:** (+)-balasubramide derivative 3C, atherosclerosis, AMPK, STAT1, STING

## Abstract

We previously reported the neuroprotective effects of (+)-balasubramide derived compound 3C, but its action on atherosclerosis *in vivo* remains unknown. The study was designed to investigate the potential effects of 3C on atherogenesis and explore the possible underlying mechanisms. 3C ameliorated high-fat diet-induced body weight gain, hyperlipidemia, and atherosclerotic plaque burden in apolipoprotein E-deficient (ApoE^-/-^) mice after 10 weeks of treatment. 3C suppressed the expression of genes involved in triglyceride synthesis in liver. 3C prevented aortic inflammation as evidenced by reduction of adhesive molecule levels and macrophage infiltration. Mechanistic studies revealed that activation of AMP-activated protein kinase (AMPK) is central to the athero-protective effects of 3C. Increased AMPK activity by 3C resulted in suppressing interferon-γ (IFN-γ) induced activation of signal transducer and activator of transcription-1 (STAT1) and stimulator of interferon genes (STING) signaling pathways and downstream pro-inflammatory markers. Moreover, 3C inhibited ox-LDL triggered lipid accumulation and IFN-γ induced phenotypic switch toward M1 macrophage in RAW 264.7 cells. Our present data suggest that 3C prevents atherosclerosis via pleiotropic effects, including amelioration of lipid profiles, vascular inflammation and macrophage pro-inflammatory phenotype. 3C has the potential to be developed as a promising drug for atherosclerosis and related cardiovascular disease.

## INTRODUCTION

Atherosclerosis is a progressive disease which arises from disturbed lipid profiles and vascular inflammation at the large artery. The basic pathological process of atherosclerosis includes migration of the inflammatory cells into the sub-endothelial area and deposition of lipids and cell debris on the vessel wall that ultimately lead to myocardial infarction or stroke which are still the leading cause of mortality and morbidity [1, 2].

Multiple risk factors participate in the process of atherosclerosis development. Dysregulation of lipid metabolism and other metabolic dysfunction such as obesity, hypertension, insulin resistance, diabetes, and disturbed hemodynamic shear stress lead to endothelial activation [3–5]. A variety of inflammatory cells such as monocytes, T-lymphocytes, mast cells, eosinophils, dendritic cells are attracted, rolling on the endothelium surface and invade into the sub-endothelial sites, upregulation of cytokines and chemokines further facilitates this process [6]. These infiltrated inflammatory cells, especially macrophages, play a pivotal role in plaque growth. Monocytes differentiate into macrophages under the inflammatory microenvironment, engulf large amounts of lipids to form foam cells, and release a large number of inflammatory factors which in turn accelerate foam cell formation. Finally, the vascular bed collapses and vulnerable plaques rupture to block blood vessels [7].

AMPK is a well-known intracellular energy sensor and regulator, which can sense the intracellular energy level when the ratio of AMP to ADP increases. Upon AMPK’s activation, intracellular anabolic pathways are restricted with the catabolic pathways activated to restore the energy balance [8]. In addition to being an energy sensor, increasing evidence has shown that AMPK also exerts anti-inflammation effects by inhibiting cytokines including interferons, tumor necrosis factors, interleukins, mediated inflammatory signaling pathways [9–12].

Janus kinases/signal transducer and activator of transcription (JAK/STAT) signaling pathway is a major intracellular inflammatory cascade that transmits the intracellular signaling to the nucleus, promoting inflammatory response, accelerating the development of atherosclerosis [13, 14]. Moreover, deletion of STAT1 attenuates the progression of atherosclerosis [15]. Thus, the JAK/STAT pathway is an attractive therapeutic target for treating inflammation-mediated vascular diseases especially atherosclerosis.

(+)-Balasubramide is an eight-membered lactam compound extracted from the leaves of the Sri Lankan plant *Clausena indica*. Previous studies have shown that clausenamide, a biosynthetic precursor of balasubramide, has anti-apoptosis [16] and anti-neuroinflammation effects, acting as an oxygen free radical scavenger and AMPK activator [17, 18]. We have previously demonstrated a central role of AMPK-STAT1 axis in vascular inflammation regulation [19]. However, the pharmacological effect of (+)-Balasubramide in atherosclerosis and the underlying mechanism remain unknown.

In the present study, we demonstrated that 3C administration potently attenuates atherosclerotic lesion burden in HFD-fed ApoE^-/-^ mice, possibly by inhibiting inflammatory response, modulating lipid metabolism and deposition, resulting in improvement of plaque stability. The mechanistic studies indicate that 3C inhibits JAK2-STAT1-STING signaling pathway through activation of AMPK. Thus, our studies have established the potential therapeutic applications of 3C as a novel atherosclerosis treatment.

## RESULTS

### Treatment of ApoE^-/-^ mice with 3C ameliorates plaque burden and progression

**Figure 1:**
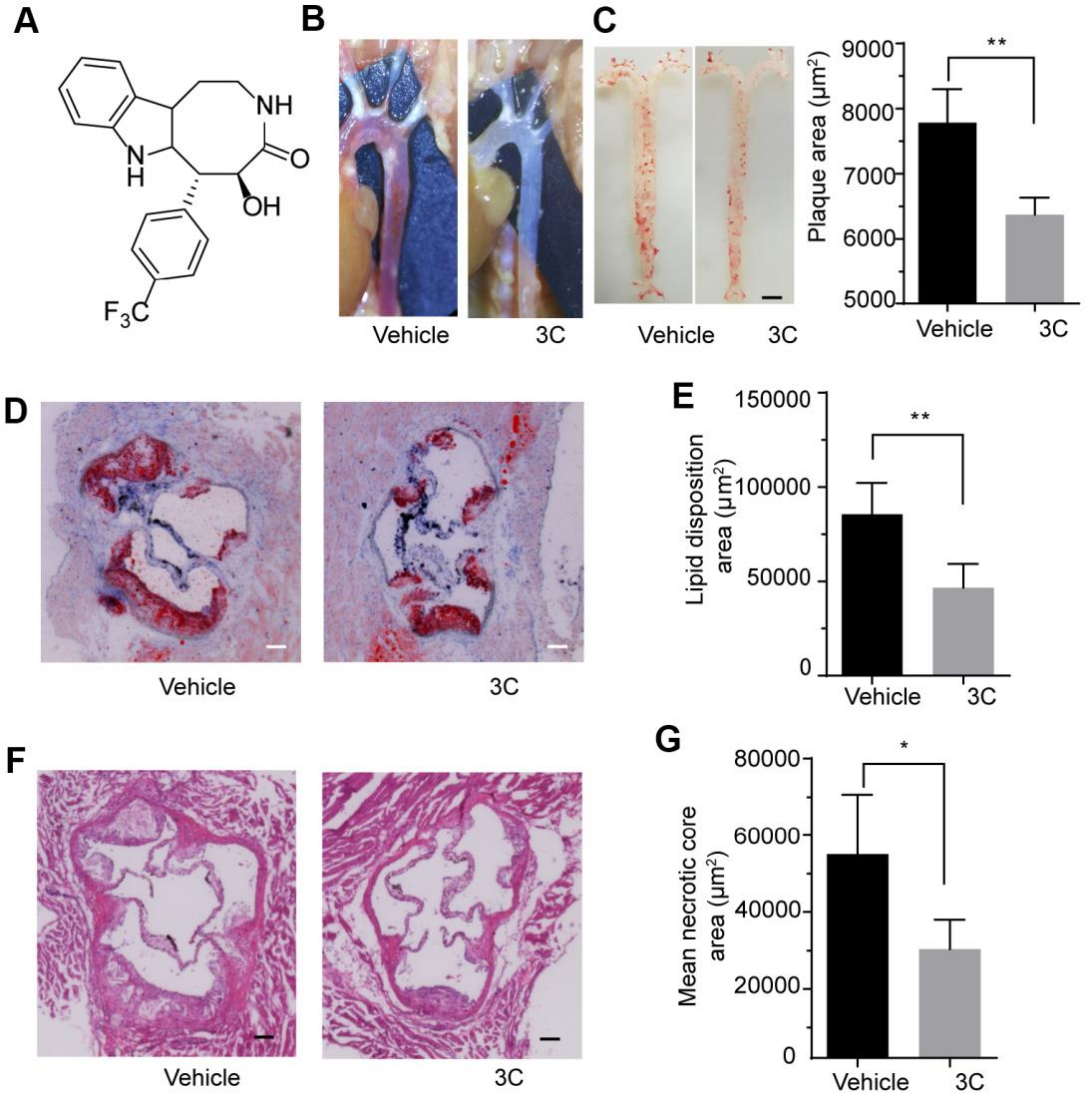
**3C protects ApoE^-/-^ mice from high-fat diet-induced atherosclerotic plaque formation.** (**A**) Chemical structure of compound 3C. (**B**) Representative images *in situ* of aortic arch and its branches with white plaques. (**C**) Representative images of the Enface staining with oil red-O of the whole aorta and quantification of Oil Red O–stained Enface aorta of plaque area for ApoE^-/-^ mice treated with vehicle or 3C. (**D**) Representative images of plaques in the aortic root after 10 weeks of HFD was determined by staining sections with Oil red O. (**E**) Quantification of Oil Red O–stained aortic root for the lipid disposition area (n=5 per group). (**F**) Representative images of plaques in the aortic root staining sections with H&E staining. (**G**) Quantification of H&E-stained aortic root for the plaque lesion area and mean necrotic area (n=5 per group). All data were assessed using Student’s *t*-test and are present as mean±SEM. *P <0.05; **P<0.01.

We first examined the efficacy of 3C (10 mg/Kg/day) in HFD-induced atherosclerosis model in ApoE^-/-^ mice. The aortas from 10 weeks HFD-fed ApoE^-/-^ mice exhibited atherosclerotic plaques that were readily visible on the inner side of the aortic arch, the bifurcation of the brachiocephalic and left common carotid arteries where exhibit disturbed blood flow and are typical atheroprone area. Treatment with 3C ameliorated the extent of plaques burden in these sites (Figure 1B). As measured by *En face* Oil-red O staining, aortas showed significantly attenuated atherosclerotic lesion areas in the aortic tree (Figure 1C). Oil Red O staining of the aortic root cryosections revealed a robust decrease of lipid disposition in atherosclerotic lesion area after 3C treatment compared to vehicle (Figure 1D, 1E). The smaller lesion size was accompanied by a reduction in necrotic core formation, evaluated using H&E staining according to established protocols [20] (Figure 1F, 1G). Taken together, these data demonstrate that 3C exerts preventative effect on atherogenesis in HFD-fed ApoE^-/-^ mice.

### 3C suppresses adhesive molecules expression in atherosclerotic lesions

**Figure 2:**
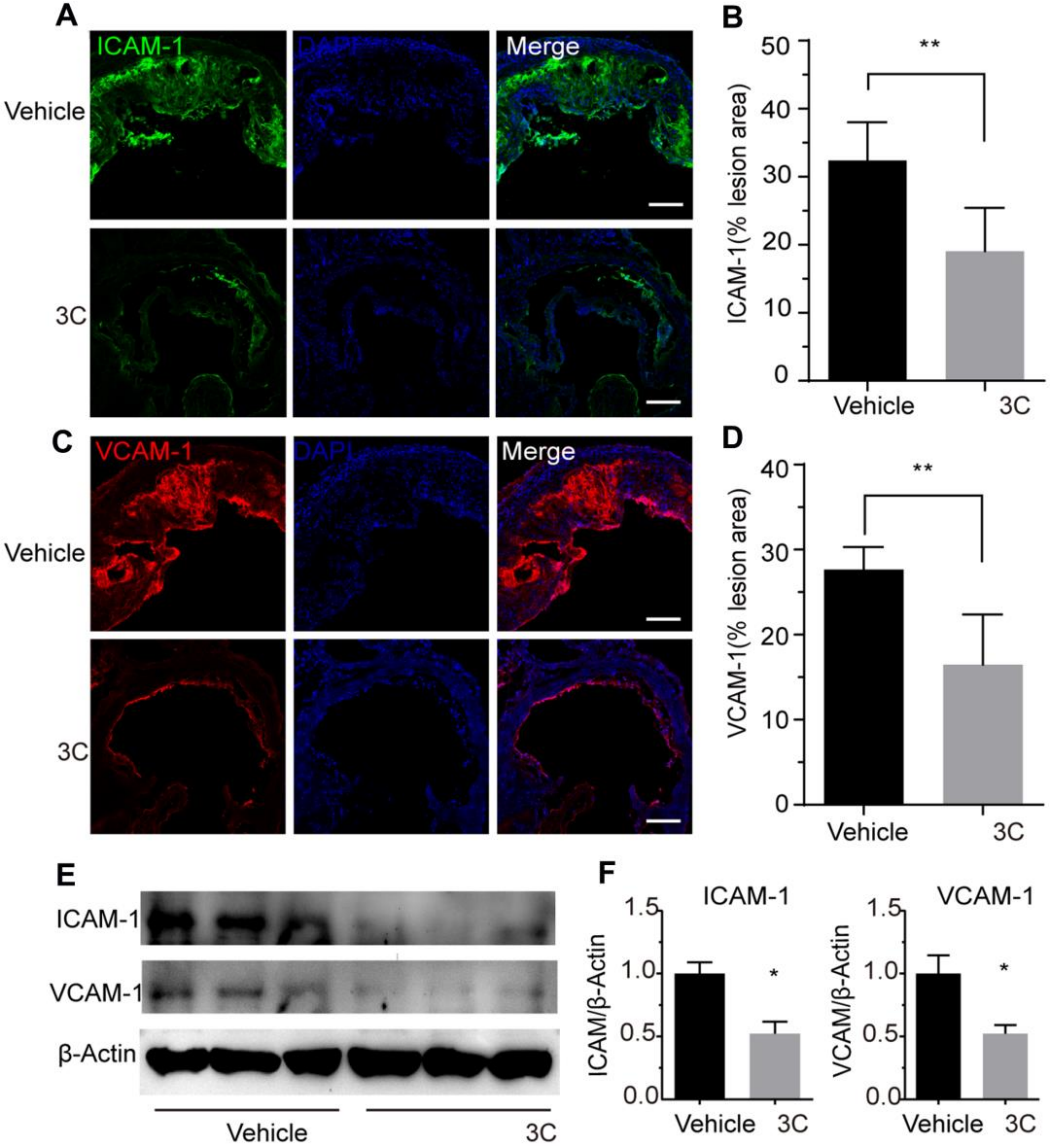
**Treatment of ApoE^-/-^ mice with 3C inhibits vascular inflammation.** (**A**) Representative images of immunofluorescence staining for ICAM-1 as an indicator of the vascular inflammation from the aortic root of ApoE^-/-^ mice treated with vehicle or 3C. (**B**) Quantification of ICAM-1 positive areas in plaques (n=6 per group). (**C**) Representative images of immunofluorescence staining for VCAM-1 form the aortic root of ApoE^-/-^ mice treated with vehicle or 3C. (**D**) Quantification of VCAM-1 positive areas in plaques (n=6 per group). (**E**) Protein expression level of ICAM-1 and VCAM-1 protein in the aorta were assessed by Western blot analysis. (**F**) Quantitative results for the relative expression level of ICAM-1 and VCAM-1 normalized to β-Actin (n=3 per group). All data were assessed using Student’s *t*-test and are present as mean±SEM. *P <0.05; **P<0.01.

Atherosclerosis is a progressive disease characterized by the process of constant accumulation of lipid in the artery wall which can be accelerated by the chronic inflammatory response. Because of the hemodynamic effects, atherosclerotic plaques prefer to form at vessel branch points, bifurcations, and regions of high curvature where display oscillatory shear stress [21]. In these atheroprone sites, endothelial cells undergo apoptosis which is accompanied by increased vascular permeability and upregulation of adhesive molecules such as ICAM-1 and VCAM-1 [22]. These adhesion molecules will recruit the circulating inflammatory cells such as monocytes, T-lymphocytes, mast cells, eosinophils, dendritic cells to the intima to engulf the lipids which is the major cause for plaque progression. To explore the involvement of adhesive molecules in the preventive effect of 3C on atherogenesis, immunostaining was performed. Treatment with 3C downregulated the ICAM-1/plaque ratio by 10% (Figure 2A, 2B) and VCAM-1/plaque ratio by 15% (Figure 2C, 2D). These observations were further confirmed by Western blotting analysis using the aortic tissues (Figure 2E, 2F). These data support the concept that 3C may mitigate vascular inflammation through downregulating adhesive molecules expression.

### 3C improves lipid profile

**Figure 3:**
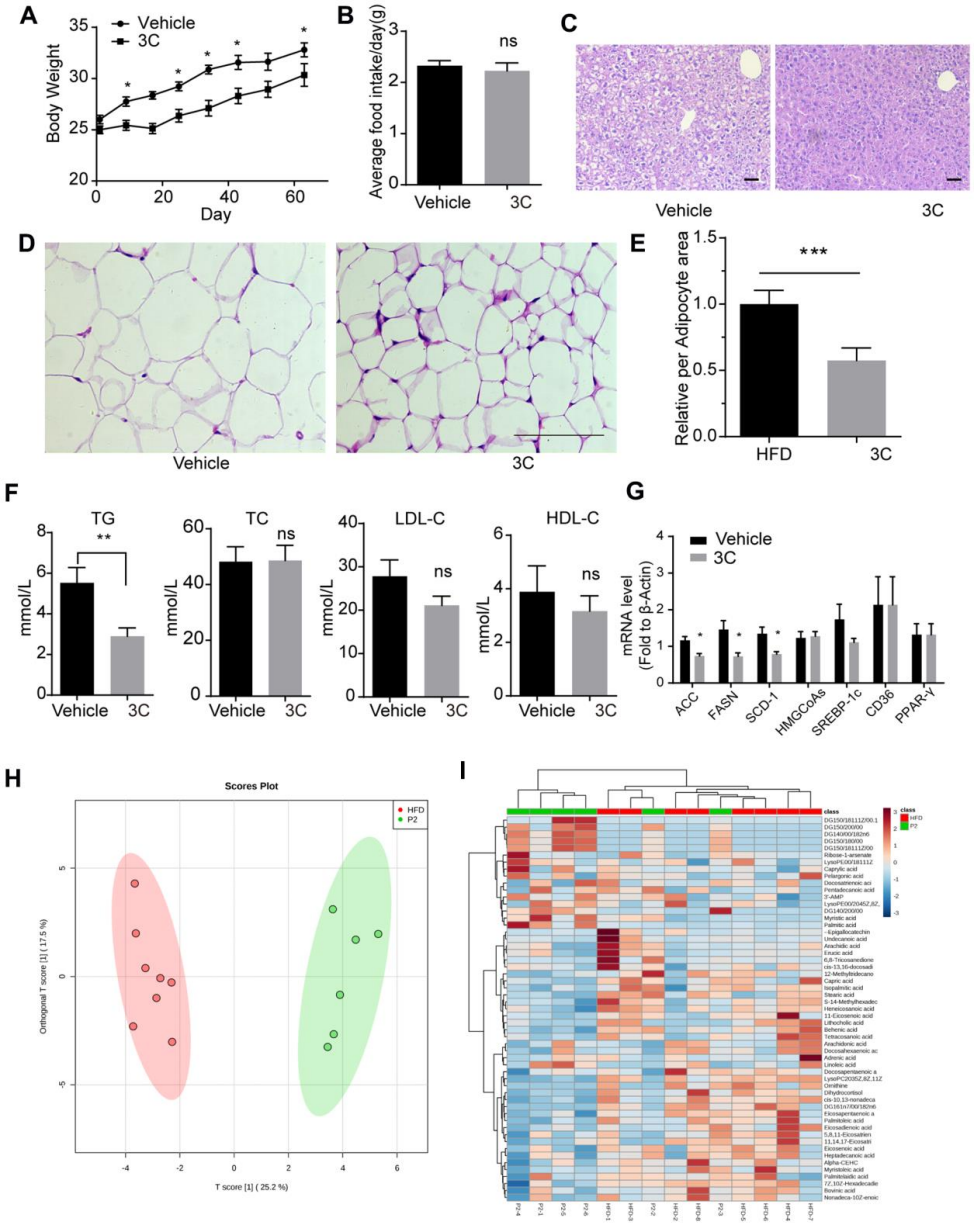
**Treatment with 3C regulates the HFD induced dysfunctional lipid profile.** (**A**) Weight gain curve of ApoE^-/-^ mice from different groups. (**B**) Average food intake of every ApoE^-/-^ mouse from different groups. (**C**) Representative images of paraffin section of the liver staining with H&E staining. (**D**) Representative images of paraffin section of epididymal fat with H&E staining. (**E**) Quantitation of relative area of per adipocyte. (**F**) Quantification of plasma levels of triglycerides, total cholesterol, low-density lipoprotein cholesterol, and high-density lipoprotein cholesterol in serum from different groups. (**G**) Fold change of ACC, FASN, SCD-1, HMGCoAs, SREBP-1c, CD36, and PPAR-γ mRNA expression level from livers of different groups (n=6 per group). (**H**) The OPLS-DA scores plot showing the groupings of 3C (also called P2, green), and vehicle (red) individuals based on their lipid profiles. (**I**) Clustered heat image map of the relationship between differential lipids and biochemical parameters. All data were assessed using Student’s *t*-test and are present as mean±SEM. *P<0.05; **P<0.01.

ApoE^-/-^ mice fed with high-fat diet exhibit accelerated atherosclerosis plaques formation and dysfunctional lipid metabolism characterized by the elevated level of triglyceride (TG), total cholesterol (TC) and LDL-C. Besides, long-term high-fat diet feeding causes weight gain and lipid accumulation in the liver and adipose tissue. Therefore we examined the lipid profile after 3C treatment in the high-fat diet fed ApoE^-/-^ mice. As shown in Figure 3A, 3C treatment caused a slight decrease in body weight. Intriguingly, food intake was not altered by 3C (Figure 3B). H&E staining for the liver showed less accumulation of lipid after the treatment of 3C (Figure 3C), reduced lipid droplet size was also observed with 3C treatment in inguinal white adipose tissues (Figure 3D, 3E). 3C significantly reduced the serum TG levels but had no obvious effects on TC, or HDL-C, even though LDL-C had a trend to be lower (Figure 3F).

Gene expression profiling in livers showed ACC, FASN, and SCD-1, which are key enzymes in TG synthesis, were dramatically downregulated with 3C treatment, but genes involved in cholesterol synthesis remained unchanged (Figure 3G). Furthermore, lipidomic analysis of the liver samples using the PLS-DA model indicated that 3C exhibited significant effects on lipid metabolism. The score plot indicated significant metabolic differences between the two groups with good model quality (R^2^ of 0.88, Q^2^ of 0.67) (Figure 3H). A total of 55 variables were chosen for the heatmap analysis. As shown in the clustered image map (Figure 3I), most of the differential metabolites showed correlations with diglyceride. DG (14:0/0:0/18:2n6), DG (14:0/20:0/0:0), DG (15:0/18:0/0:0), DG (15:0/18:1(11Z)/0:0), DG (15:0/20:0/0:0) and DG (16:1n7/0:0/18:2n6) were significantly increased. Diglyceride has been shown to exert anti-obesity effect which is correlated to cardiovascular disease [23, 24] and may explain the body weight loss with 3C treatment in the current study.

### 3C inhibits macrophage activation and foam cell formation

**Figure 4:**
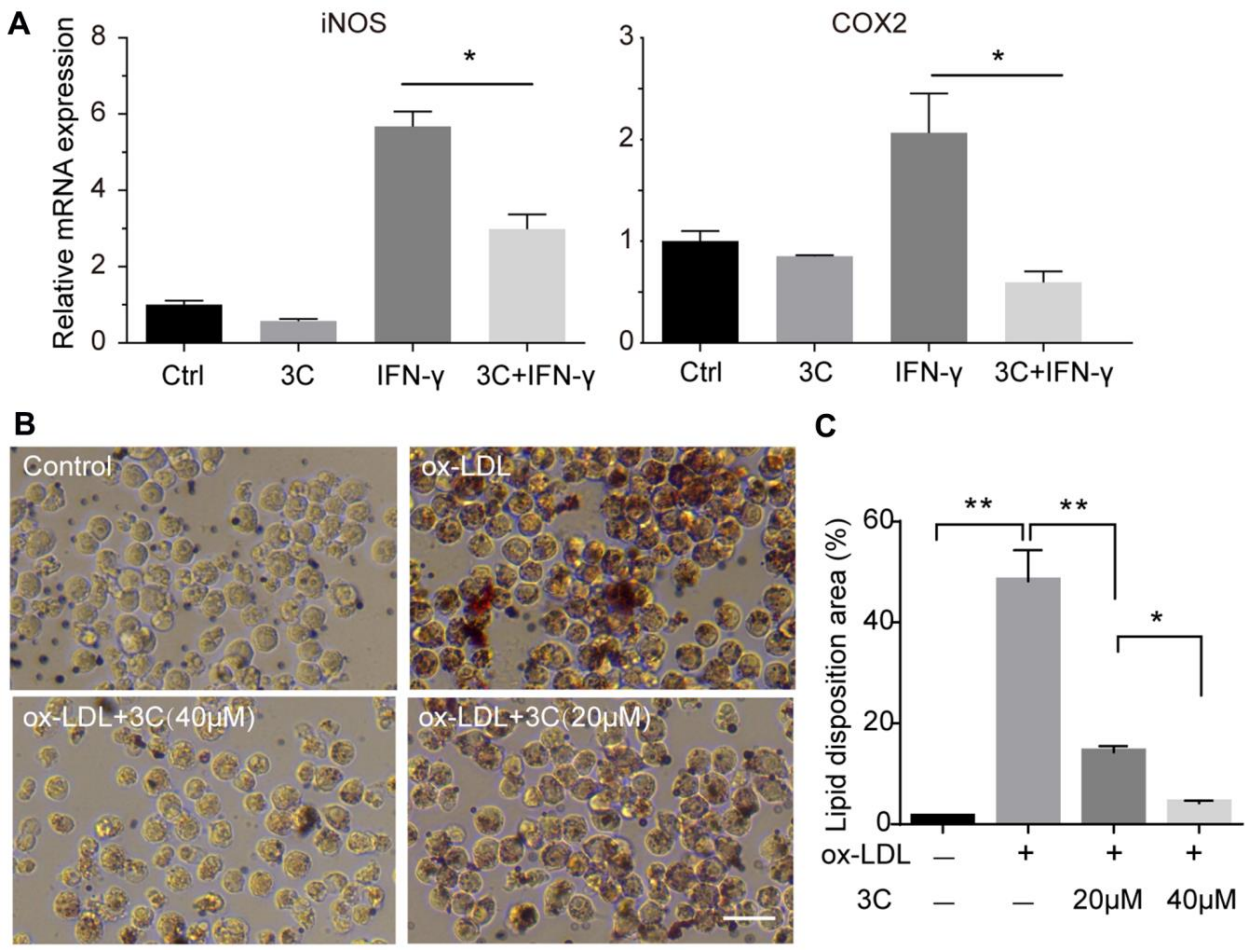
**3C inhibits macrophage polarization and foam cell formation.** (**A**) Fold change of iNOS, COX-2 and CD206 mRNA expression level. β-Actin was used for sample loading normalization. (**B**) 3C dose-dependently attenuate ox-LDL induced foam cell formation. (**C**) Quantification of the lipid disposition area versus the cellular area. All experiments were performed at least three times; were assessed using Student’s *t*-test and are present as mean±SEM. *P<0.05; **P<0.01.

Migration of monocytes to the endothelium and differentiation into M1 macrophages under the atherosclerotic inflammatory microenvironment are key events in plaque initiation [25]. Macrophage engulfs lipid deposited in the sub-endothelial area, resulting in foam cell formation and accelerating the growth of plaques [26, 27]. IFN-γ is also a pro-inflammatory cytokine that can stimulate polarization [28] and accelerate foam cell formation [29]. So, we next examined the effect of 3C on macrophage polarization and foam cell formation. Treatment of RAW264.7 cells with 3C significantly decreased the levels of M1 macrophage markers (Figure 4A). Foam cell formation is a critical step in atherogenesis, exposure to ox-LDL (100μg/ml, 24h) markedly increased lipid disposition in RAW264.7 cells, while pretreatment with 3C dose-dependently decreased lipid levels in macrophages (Figure 4B, 4C).

### 3C suppresses JAK2/STAT1 signaling via AMPK activation

**Figure 5:**
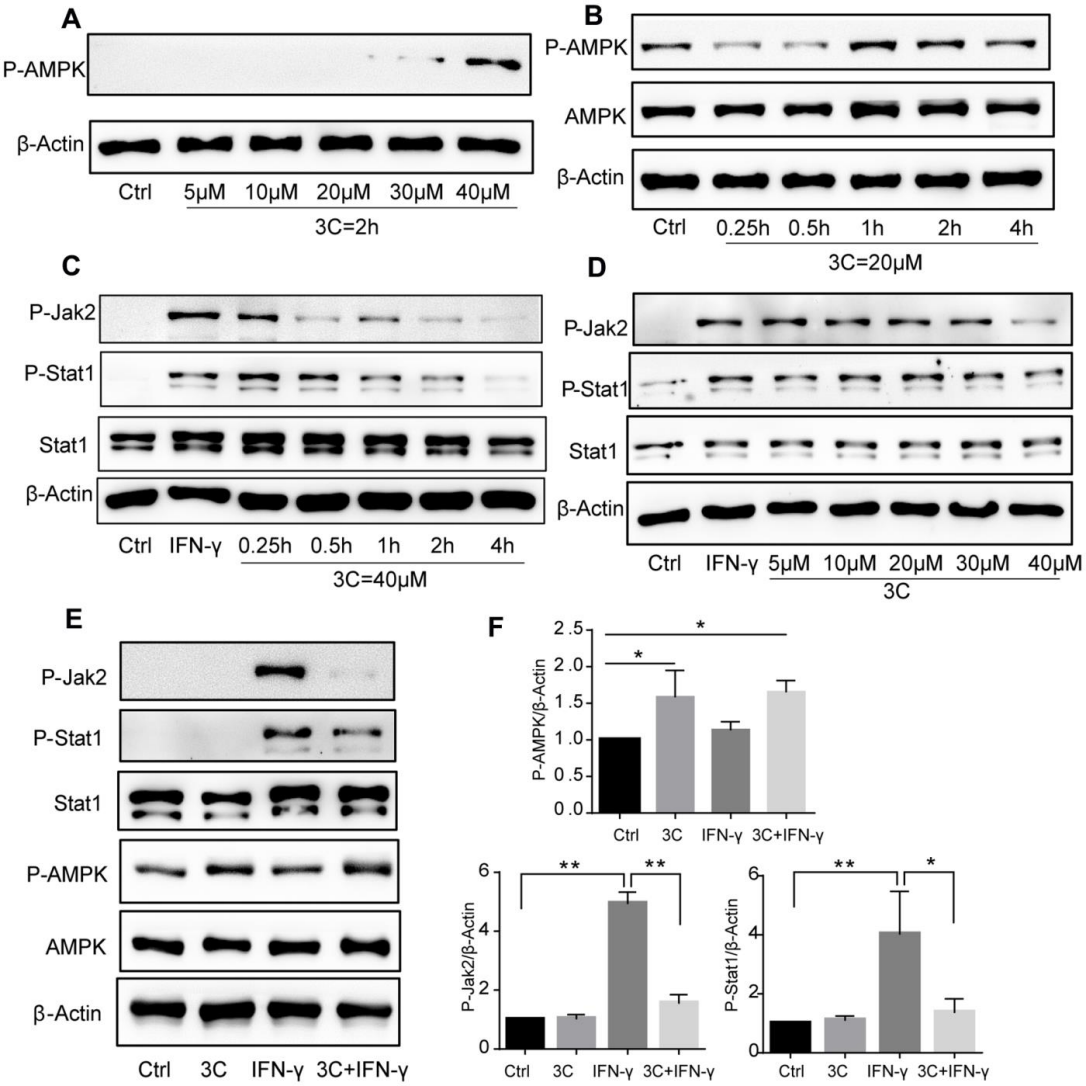
**3C induces AMPK phosphorylation and inhibits JAK2-STAT1 signaling.** (**A**) 3C dose-dependently induced AMPK phosphorylation. (**B**) 3C time-dependently induced AMPK phosphorylation. (**C**) 3C time-dependently inhibit IFN-γ induced JAK2 and Stat1 phosphorylation. (**D**) 3C dose-dependently inhibit IFN-γ induced JAK2 and Stat1 phosphorylation. Cells were pre-treated at indicated different time or with different concentrations of P2 for 4h followed by 10ng/mL IFN-γ incubation for 30min then the cell was harvested. (**E**) 3C activate AMPK and inhibit IFN-γ stimulated JAK2-Stat1 signaling but IFN-γ has no effect on AMPK. (**F**) Relative level of P-AMPK, P-JAK2 and P-Stat1 normalized to β-Actin (n=3 per group). All experiments were performed at least three times; were assessed using Student’s *t*-test and are present as mean±SEM. *P<0.05; **P<0.01.

We have previously demonstrated that activation of STAT1 by PDGFRβ signaling drives vascular inflammation and atherogenesis [20], activation of AMPK suppresses STAT1 activity [19]. Also, STAT1 was found to be involved in macrophage apoptosis induced by endoplasmic reticulum stress and foam cell formation [15, 30]. To explore the mechanism by which 3C prevents atherosclerosis development, we hypothesized that 3C might suppress JAK2/STAT1 signaling through activating AMPK. Firstly, phosphorylation of AMPK at Thr172 was dose- and time-dependently increased upon compound 3C stimulation (Figure 5A, 5B). As shown in Figure 5C, 5D, 3C inhibited IFN-γ, which is a classical macrophage activator, induced JAK2-STAT1 activation in a dose- and time-dependent manner. Consistent with our previous observations [17], JAK2-STAT1 axis was inhibited by compound 3C accompanied by the activation of AMPK (Figure 5E, 5F) upon short-time exposure to IFN-γ in RAW264.7 cells.

### 3C inhibits JAK2/STAT1 and downstream signaling

**Figure 6:**
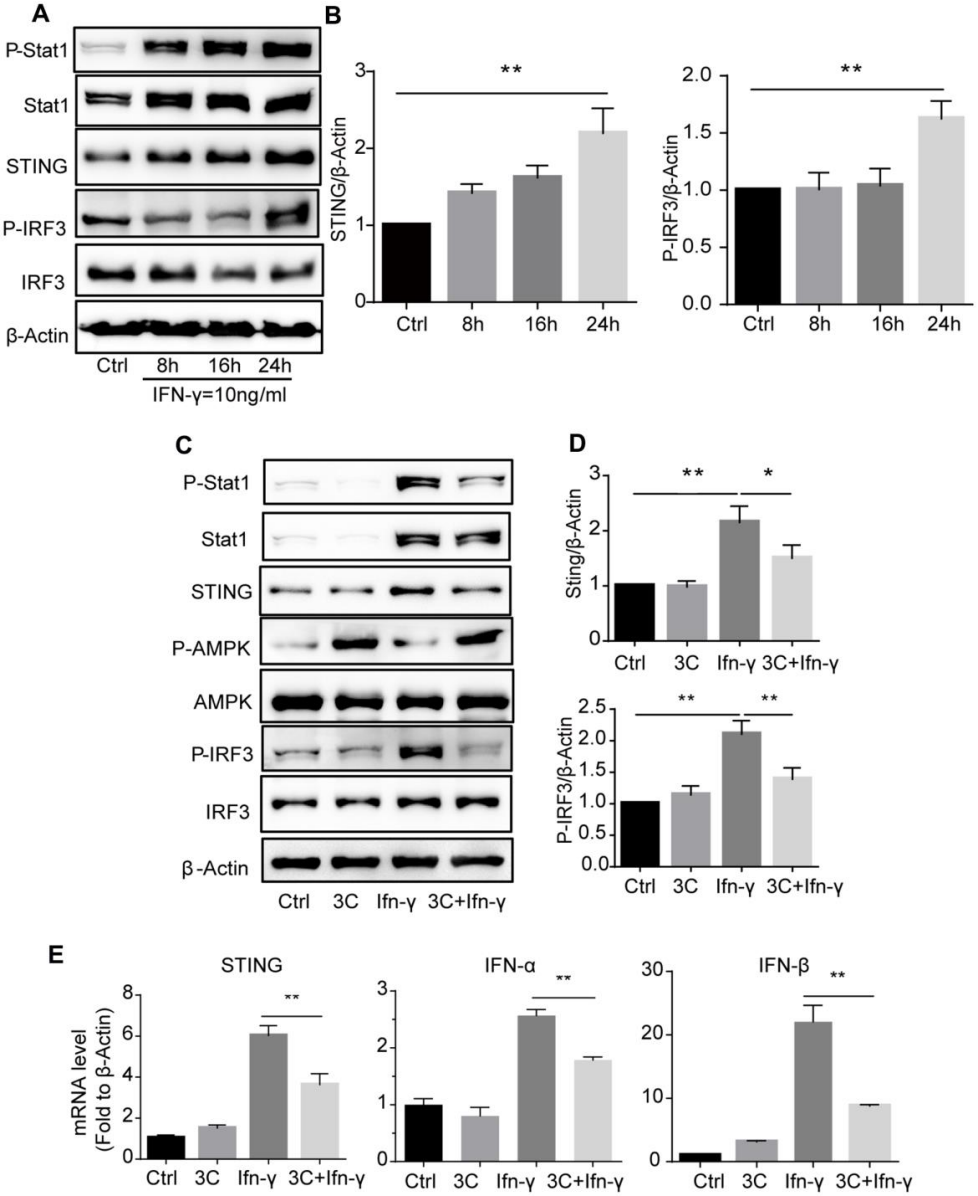
**3C inhibits IFN-γ stimulated JAK2-STAT1 signaling and downstream signaling in AMPK dependent manner.** (**A**) IFN-γ time-dependently induce phosphorylation of Stat1 and initiate downstream STING-IRF3 signaling. (**B**) Quantitative results for the relative level of STING and P-IRF3 normalized to β-Actin (n=3 per group). (**C**) 3C activate AMPK and inhibit IFN-γ stimulated Jak-Stat1 and downstream STING-IRF3 signaling. (**D**) Relative level of STING, P-IRF3 normalized to β-Actin (n=3 per group). (**E**) Relative gene expression of STING, IFN-α, IFN-β normalize to β-Actin. Results are expressed as means ± SEM (n = 3). *P<0.05, **P<0.01.

JAK2/STAT1 activation upon interferon or PDGF stimulation induces expression of IFN-stimulated genes (ISGs) and the release of chemokines [31]. To further investigate the downstream signaling, we analyzed the IFN-γ primed macrophages and found that long-time exposure to IFN-γ drastically induced the expression of STAT1 and STING and the downstream IRF activation (Figure 6A, 6B). Treatment with 3C activated AMPK and markedly attenuated STAT1 phosphorylation and STING-IRF3 activation (Figure 6C, 6D). Gene expression analysis with qRT-PCR also showed that STING target genes including IFN-α, IFN-β was down-regulated (Figure 6E). However, other cytokines including TNF-α, IL-6 and IL-1β remained unchanged (Supplementary Figure 1A).

### Inhibition of JAK2/STAT1 and downstream signaling with 3C is AMPK dependent

**Figure 7:**
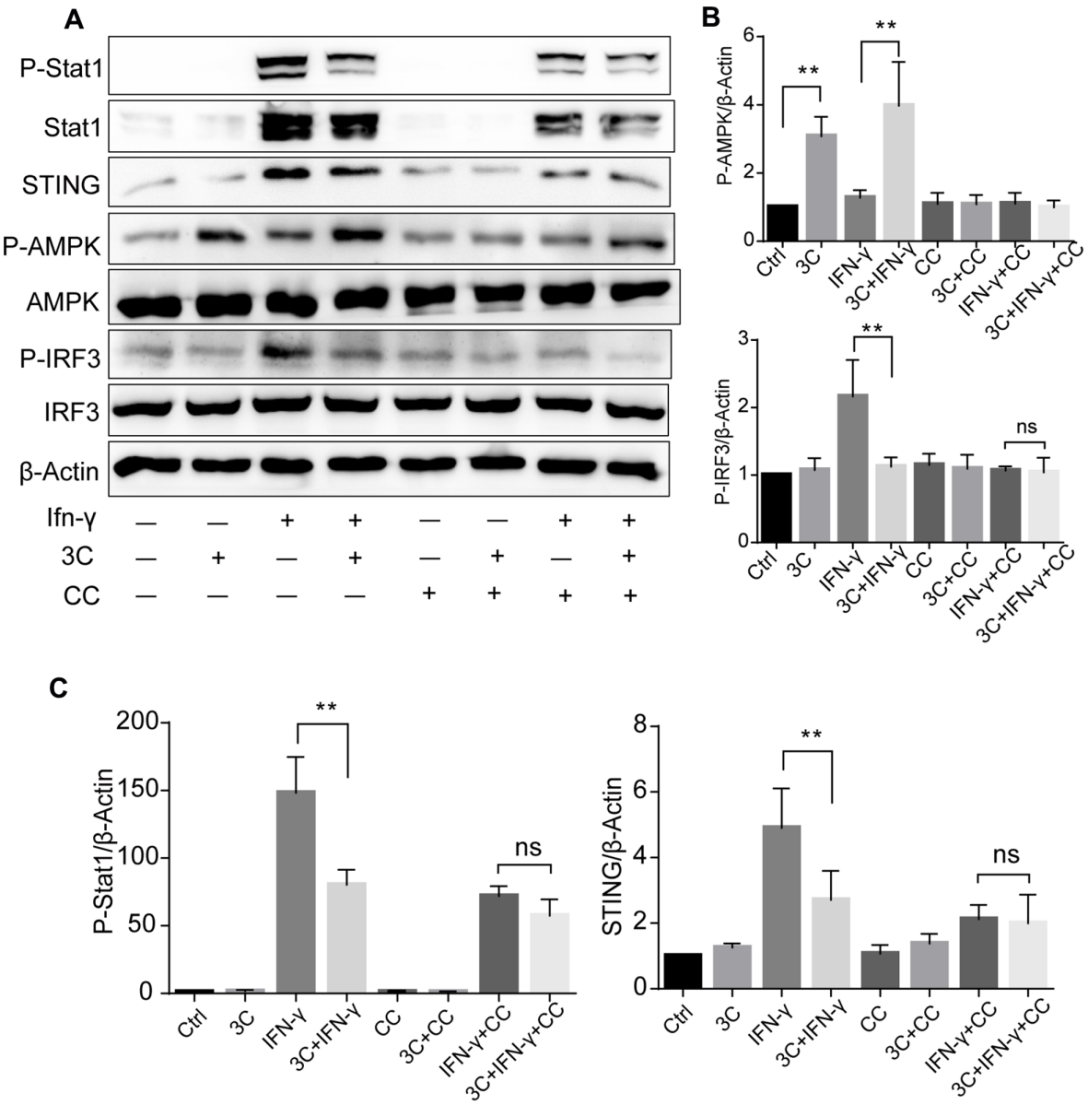
**3C inhibits JAK2/STAT1 and downstream signaling in AMPK dependent manner.** (**A**) AMPKα inhibition with compound C attenuate the suppressive effect of 3C on STING-IRF3 signaling. (**B**, **C**) Quantification of the relative level of P-Stat1, P-AMPK, STING and P-IRF3 normalized to β-Actin (n=3 per group). The experiments were performed in triplicate and repeated at least three times on different days. Results are expressed as means ± SEM (n = 3). *P<0.05, **P<0.01.

To gain further insight into whether these observations are AMPK dependent, we pretreated the cells with a potent AMPK inhibitor Compound C (CC). As depicted in Figure 7A–7C, Compound C abolished the effects of 3C on AMPK activation as well as STAT1 and STING-IRF3 signaling inhibition, indicating that the inhibitory effects of 3C on activation of JAK/STAT1 and downstream STING-IRF3 are AMPK dependent.

### 3C treatment activates AMPK and inhibits STAT1-STING signaling in ApoE^-/-^ mice

**Figure 8:**
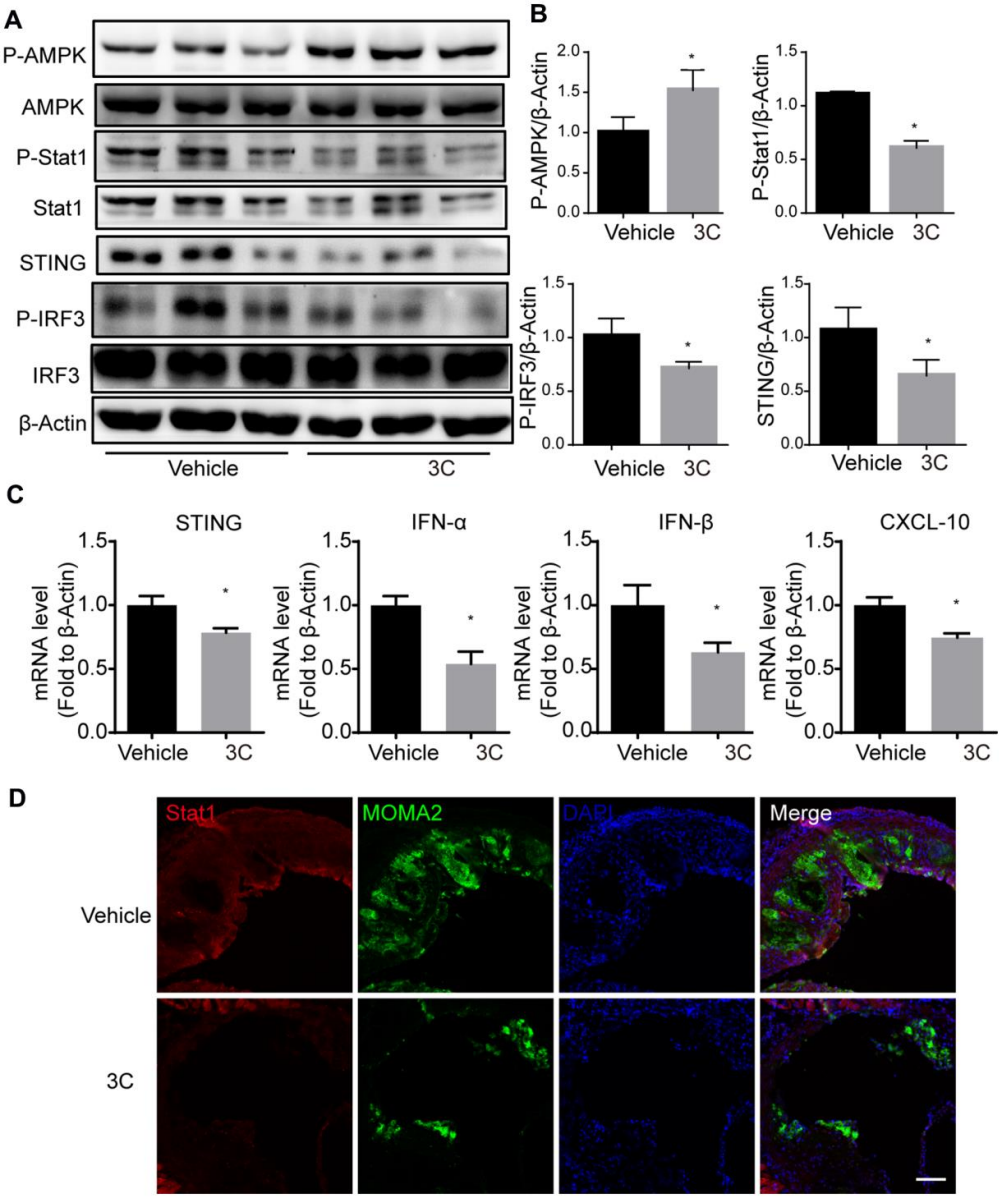
**Treatment of ApoE^-/-^ mice with 3C inhibits JAK-STAT1 and downstream signaling.** (**A**) Relative level of P-Stat1; Stat1; P-AMPK; AMPK; STING; P-IRF3 and IRF3 was assessed by Western Blot (n=3 per group). (**B**) Quantitative results for the relative expression level of P-Stat1; P-AMPK; STING and P-IRF3normalized to β-Actin (n=3 per group). (**C**) Fold change of STING, IFN-α, IFN-β, and CXCL10 mRNA expression level from aortas of different groups. (**D**) Representative images of immunofluorescence staining for Stat1 from the aortic root of ApoE^-/-^ mice treated with vehicle or 3C. All data were assessed using Student’s *t*-test and are present as mean±SEM. *P<0.05.

To extend our *in vitro* observations and establish the molecular mechanism behind the preventative effects of 3C in atherogenesis, we further evaluated the AMPK activation and STAT1-STING signaling in the aortas of ApoE^-/-^ mice. 3C activated AMPK, which was accompanied by reduction of STAT1 and IRF3 phosphorylation (Figure 8A, 8B), as well as levels of downstream inflammatory genes expression such as IFN-α, IFN-β, and CXCL10 (Figure 8C). Intriguingly, we also observed that TNF-α; IL-6; MCP-1, and IL-1β were differentially expressed in the aorta samples after 3C treatment (Supplementary Figure 1B). As a result, 3C treatment dramatically reduced STAT1 expression and macrophage infiltration into the atherosclerotic plaques, as evidenced by the immunofluorescence staining of MOMA 2 shown in Figure 8D.

## DISCUSSION

Atherosclerosis is a progressive vascular occlusive disease caused by multiple factors. Over the past twenty years, the use of statins and lipid-lowering therapy have led to the decrease of mortality and morbidity of atherosclerotic cardiovascular diseases, but they still rank the top cause of death world wide. [32]. Thus considerable attention has been paid to find the therapeutic target and treatment for atherosclerosis.

A series of evidence has indicated that AMPK activation markedly attenuates atherosclerosis development. AMPK is an intracellular sensor of energy metabolism that regulates multiple physiological processes, including lipid and glucose metabolism. Once AMPK is activated, pathways directing catabolism are switched on and pathways controlling anabolism are shut down. *In vivo* studies have shown that genetic or pharmacological activation of AMPK decreases lipid biogenesis and accumulation in the liver through inhibition of key enzymes and transcription factors including ACC, FAS, and SREBP1 [33–35]. Moreover, AMPK activation with small molecule activators can enhance reverse cholesterol transport by upregulating expression of ABCA1, ABCG1, and SR-BI in ApoE^-/-^ mice [36]. In addition to regulation of energy metabolism, our previous studies have shown that AMPK activation also suppresses inflammatory response [19]. Moreover, activation of AMPK is also beneficial to the maintenance of vascular permeability and can block vascular inflammatory processes such as phenotypic transition of smooth muscle cells and macrophages [37]. All these observations strongly indicate that AMPK is an emerging therapeutic target for treatment of metabolic syndrome and cardiovascular diseases.

In the present study, we demonstrated that compound 3C, a naturally derived compound potently decreased the atherosclerotic plaques burden in HFD fed ApoE^-/-^ mice. Plaques in the aortic root, arch, and bifurcations were significantly reduced after the 3C treatment. Further examinations showed that 3C treatment reduced lipid deposition and adhesive molecules ICAM-1/VCAM-1 expression in the plaque area. In the atheroprone area, upregulation of adhesion molecules and lipid deposition especially the oxidized low-density lipoprotein drive the growth of plaques. In the present study, the decreased expression of ICAM-1 and VCAM-1 may result in fewer monocytes migration and infiltrate into the subintimal site. The advanced plaques containing less collagen and fewer vascular smooth muscle cells but have larger necrotic cores and thinner fibrous cap are vulnerable to rupture to form thrombosis. In this study we also observed that 3C decreased necrotic core area in addition to the preventative effect of plaque progression, indicating 3C regulates both plaque progression and stability.

Hyperlipidemia is an important risk factor in the development of atherosclerosis. Currently, most clinically used lipid-lowering therapies are based on lowering cholesterol, especially the level of low-density lipoprotein cholesterol. Recent studies have found that high level of triglyceride is a strong and independent risk factor for atherosclerotic cardiovascular disease (ASCVD). Analysis of serum lipid profile revealed a significant decrease in triglyceride levels after 3C administration, but total cholesterol, low-density lipoprotein cholesterol, and high-density lipoprotein cholesterol remained unchanged. Gene expression profiling suggested that 3C decreased the key enzymes of fatty acid synthesis and glyceride synthesis in ApoE^-/-^ mice livers, resulting in alleviation of lipid deposition. ApoE^-/-^ mice treated with 3C gained less body weight and had smaller lipid droplet size. Further investigation of the lipid composition in livers with lipidomics revealed the significant elevation of diglyceride levels. Diglyceride has been shown to reduce both fasting and postprandial blood TG levels, as well as glycosylated hemoglobin levels in type 2 diabetes, and prevent fat accumulation in animals and humans [23, 24]. Furthermore, previous studies reported that fasting-induced AMPK activation inhibits DGAT activity, which is an enzyme that catalyzes the formation of triglycerides from diacylglycerol and Acyl-CoA. [38]. Taken together, our results suggest that modulation of lipid metabolism is attributable to the athero-protective effects of 3C.

Macrophage is an important element in inflammatory response and is considered to accelerate the pathogenesis of atherosclerosis under the complex vascular inflammatory microenvironment. Accelerated plaque formation by M1 macrophages mediated inflammation has attracted lots of attention. Several studies have shown that AMPK activation with specific activators such as AMP and AICAR can facilitate the transition to anti-inflammatory M2 phenotype and maintain intracellular cholesterol homeostasis [36, 39, 40]. Consistent with this, myeloid-specific deletion of AMPKα1 accelerates atherosclerosis in *Ldlr* deficiency mice [41]. IFN-γ was reported previously to synergize with LPS to promote conversion to M1 phenotype and also accelerate plaque by enhancing foam cell formation and recruitment of immune cells [42–45]. In support of this mechanism, we have demonstrated that 3C exerted favorable pharmacological activity to switch IFN-γ primed RAW264.7 cells toward M2 macrophages, suggesting modulation of macrophage phenotypic switch by 3C could play another critical role in inhibiting inflammation and preventing atherogenesis.

STING was originally identified as a cytosolic DNA sensor which can be activated by cGAMP generated by cGAS upon the detection of cytosolic DNA. Upon STING activation, TBK1 is recruited and downstream transcription factors such as IRF3, and NF-κB will also be activated to initiates type I interferon response [46–49]. Recent studies have found that gain-of-function mutation of STING leads to vasculopathy [50, 51] and apoptosis-derived membrane vesicles in SLE serum to induce type I interferon production through activation of the cGAS–STING pathway [52]. STING deficiency mice show improved metabolic outcomes upon HFD challenging [53, 54]. In this study, we demonstrated that 3C inhibited IFN-γ stimulated STING expression and the type I interferon response, which was validated in both *in vitro* and *in vivo*. Compound 3C did not affect the expression of TNF-α; IL-6 and IL-1β in IFN-γ stimulated RAW264.7 cells, but downregulated the levels of TNF-α; IL-6; MCP-1 and IL-1β in the aortas of ApoE null mice. The underlying cause for this inconsistency between *in vivo* and *in vitro* results may be explained by that the *in vivo* microenvironment is more complex and other risk factors (hyperlipidemia; low shear stress etc.) could also contribute to the development of atherosclerosis. Thus, activation of AMPK with the compound 3C may also exert beneficial effects against these “risk factors”. The other interpretation is that the STAT1 dependent but STING independent signaling is also involved in the atherosclerosis protective effect of the compound 3C *in vivo*.

**Figure 9:**
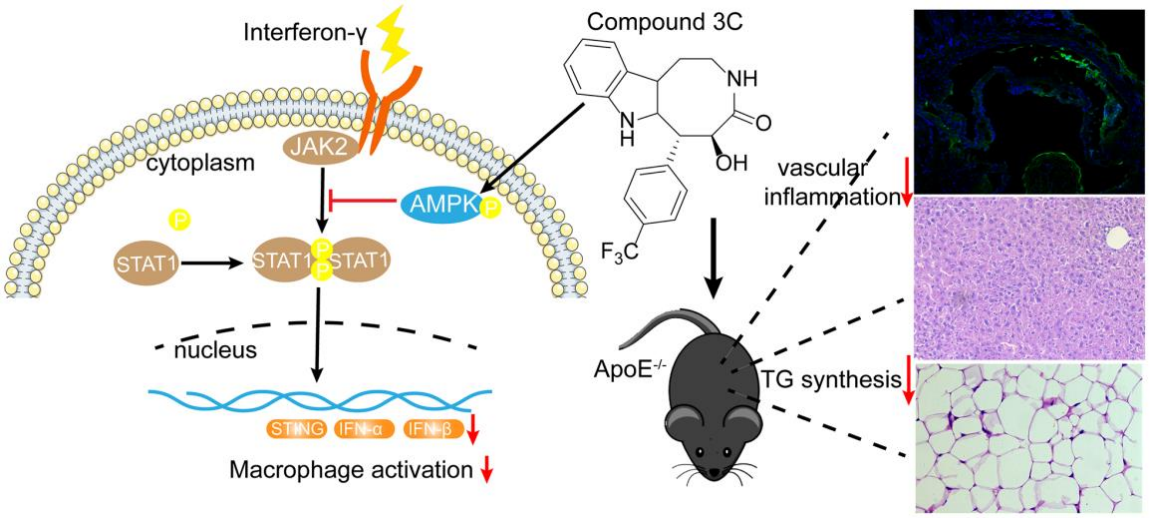
**A mechanistic schematic diagram showing Compound 3C alleviates atherosclerosis by multiple mechanisms.** Compound 3C improves lipid profile in high-fat diet-fed ApoE^-/-^ mice. Compound 3C is a potent activator of AMPK and inhibit JAK2/STAT1/STING signaling.

The present study mainly focused on the downstream effects of compound 3C on AMPK activation. Results showed that natural product-derived 3C inhibited transcription factor STAT1 activation and reduced the expression of expression of STING (Figure 9). When AMPK was inhibited by compound C these effects were abolished, indicating the anti-inflammatory effect of 3C relies on AMPK activation. There are two known upstream kinases i.e. calmodulin-dependent protein kinase kinase β (CaMKKβ) and liver kinase B1 (LKB1) that activate AMPK. Our previous study demonstrated that compound 3C increased CaMKKβ phosphorylation but has no effect on LKB1 activation in mouse primary microglia cells and BV2 cells [17]. But whether 3C activates AMPK in the present study through CaMKKβ needs further study. Currently, researches on STING activator or inhibitor are based on direct interaction of STING and the compound [55, 56]. Whether 3C has direct interaction with STING warrants further investigation.

In summary, this study explored the preventative effects of the natural product derived compound 3C on atherosclerosis. Because AMPK activation is central to the protective activity of 3C in atherosclerosis, the AMPK-STAT1-STING pathway may be a valid pharmacological target for treating inflammation-related diseases such as atherosclerosis, insulin resistance, and cardiovascular disease. Our studies imply 3C may have the potential to be developed as a promising drug for atherosclerosis and related cardiovascular disease.

## MATERIALS AND METHODS

### Reagents

Compound 3C was synthesized and provided by Dr. Hansen Lin, Guangdong Pharmaceutical University, and the purity of compound 3C was about 96%. IFN-γ was obtained from Peprotech. Compound C was purchased from APExBIO. Fludarabine was purchased from Selleck. Trizol reagent was purchased from Sigma Aldrich (St. Louis, USA). The HifairTMII1st Strand cDNA Synthesis SuperMix for qPCR and qPCR SYBR Green Master Mix were purchased from Yeasen Biotech (Shanghai, China). Antibodies for Western Blot against phospho-Jak2, phospho-Stat1, Stat1, phospho-IRF3, IRF3, phospho-AMPK, AMPK, STING, β-actin were purchased from Cell Signaling Technology (Beverly, MA, USA). Primary antibodies were used at 1:1,000. Horseradish peroxidase-conjugated secondary antibodies (Fcmacs Biotech Co. Ltd, Nanjing, China) were used at 1:5,000.

### Animal treatment

Male 8-week old ApoE^-/-^ mice were obtained from the Nanjing Biomedical Research Institute (Nanjing, China). Mice were housed in a specific pathogen-free (SPF) animal room on a 12 h circadian cycle. After 7 days of adaption, the mice were fed a high-cholesterol atherogenic Western diet (21% fat and 1.25% cholesterol, TROPHIC Animal Feed High-tech Co., Ltd, Nantong, China). 3C was administrated intraperitoneally at 10 mg/kg once daily. 10 weeks later, mice were euthanized with CO_2_, tissues and blood samples were harvested and stored at -80° C for subsequent histological evaluation and gene expression analysis. All animal experiments were performed under approval from the Institutional Animal Care and Use Committee of the China Pharmaceutical University.

### Cell culture

RAW264.7 cells were obtained from ATCC and supplied in Dulbecco’s Modified Eagle’s medium (DMEM, Gibco) supplemented with 10% fetal bovine serum (FBS; Biological Industries) and antibiotics (100 UI/mL penicillin and 100μg/mL streptomycin; Gibco) and were maintained in a humidified 5% CO2 atmosphere at 37° C.

### Serum lipid analysis

Mice were fasted overnight, and retro-orbital sinus blood was collected, allowed to coagulate for 2h, and centrifuged to isolate the serum. The serum was immediately frozen and stored at -80° C. The serum lipid profile, including total cholesterol, triglycerides, LDL cholesterol, and HDL cholesterol, was determined using commercially available kits from Bioengineering Institute (Nanjing, China).

### Fatty acid analysis

Fatty acid analysis was performed as described previously [57]. In brief, 30mg of liver tissues were homogenized with methanol. The lysates were incubated at 4° C overnight. After centrifugation, an aliquot of 400μL of supernatant was transferred to an Eppendorf microcentrifuge tube and centrifuge 10min at 18000 rpm and an aliquot of 60μL of supernatant was used for further analysis. The set-up parameters for the UPLC-QTOF-MS analysis were as follows. A BEH C18 (2.1 mm × 100 mm, 1.7μm) chromatographic column was used for separation with column temperature set at 37° C. The elution solvents were water (A) and acetonitrile/isopropyl (v/v= 80/20, B) with a flow rate of 400μL/min. The initial gradient was 70% B and kept for 2 min; increased to 75% B in 3 min; increased to 80% in 5 min; increased to 90% in 3 min; increased to 99% in 3 min and kept at 99% for 5 min before switching back to initial condition. The MS was operated at a negative electrospray ionization mode with a capillary voltage of 2.5 kV. The sample cone and the extraction cone were set at 55 V and 4 V, respectively. The source temperature was set to 150° C, and the desolvation temperature was set to 450° C with a desolvation gas flow rate of 650 L nitrogen per hour.

For statistical analysis, the raw data produced by UPLC-QTOF-MS were initially processed with Progenesis® QI (Waters Corp, Milford, MA) to detect peak signals, obtain calibration equations, and calculate the concentration of each FFA. The identification of the metabolites was compared with standards. Manual examination and correction were carried out to ensure data quality. To evaluate the similarities/differences of FFA profiles between the control and 3C groups in the cross-sectional study, a supervised multivariate model called orthogonal partial least square discriminant analysis (OPLS-DA) was built based on their overall metabolic profiles.

### Western blot analysis

Western blot analysis was performed with specific antibodies as previously described [20, 58]. The aortic tissues and cultured cells were lysed with lysis buffer containing protease inhibitor cocktail and phosphatase inhibitors. Aliquots of 20 μg total protein were separated on 11% SDS–PAGE gels and transferred to nitrocellulose membrane (Milipore). The membranes were probed with primary antibodies followed by the appropriate horseradish peroxidase-conjugated secondary antibodies.

### RNA isolation and qRT–PCR

For determining the level of gene expression, total mRNA was extracted from the cultured cells or tissues with Trizol reagent (Invitrogen, Grand Island, NY). 1 μg of RNA was reverse-transcribed into cDNA using cDNA synthesis kit following standard protocols. Expression levels of indicated genes were determined by using the specific primer as follows. Quantifications were performed by a comparative method (2^-ΔΔ^Ct) using β-actin transcripts as an internal control (Table 1).

**Table 1:** Information of the qPCR primer sequences.

**Gene name**	**Forward**	**Reverse**
ACC	TCTATCCGTCGGTGGTCTTAT	GAACATAGTGGTCTGCCATCTTA
FASN	CTCATTGGTGGTGTGGACAT	TTGGAGAGATCCTTCAGCTTTC
SCD-1	AGAAGACATCCGTCCTGAAATG	CAGCAGGACCATGAGAATGAT
HMGCoAS	GGTGGATGGGAAGCTGTCTA	ACATCATCGAGGGTGAAAGG
SREBP-1c	GAGAGCTTCTCTTCTGCTTCTC	CACGGACGGGTACATCTTTA
PPARγ	CCCTGGCAAAGCATTTGTATG	GGTGATTTGTCCGTTGTCTTTC
CD-36	GCGACATGATTAATGGCACAG	GATCCGAACACAGCGTAGATAG
iNOS	CCCAGAGTTCCAGCTTCTGG	CCAAGCCCCTCACCATTATCT
Cox-2	TGGTGCCTGGTCTGATGATG	GTGGTAACCGCTCAGGTGTTG
STING	CCCAACATTCGATTCCGAGATA	CTCGTAGACGCTGTTGGAATAA
IFN-α	GAGCACCAGAGTCAGTTTCTT	GCAGATCATCCCTTCTCCTTT
IFN-β	GGAAAGATTGACGTGGGAGAT	CAGGCGTAGCTGTTGTACTT

### Histological evaluation of atherosclerotic lesions

ApoE^-/-^ mice were sacrificed and perfused as described previously [20]. For *Enface* staining, the adventitia was thoroughly cleaned and then longitudinally cut open and pinned on the parafilm, fixed with 4% paraformaldehyde. En face pinned aortas were stained with Oil Red O and photographed by a camera. For analysis of the atherosclerosis plaque burden in the aortic root, the hearts were fixed in 4% paraformaldehyde and embedded in OCT compound. The aortic roots were serially sectioned into 8-μm sections. Oil Red O staining and H&E staining were used to evaluate the level of lipid disposition and area of atherosclerotic plaque. For cross-sectional quantification of plaque progression, frozen sections from the indicated sites of the aorta were stained with Hematoxylin and Eosin. Plaques were imaged with Leica microscope system. Necrotic core was quantified as the unstained area of plaque post Eosin staining. Fibrous cap thickness was quantified by choosing the largest necrotic core in a section and measuring the thinnest part of the fibrous cap.

### Immunofluorescent staining

The aortic roots were fixed in 4% paraformaldehyde, embedded in OCT, and cryosectioned at 8 μm. Sections were stained with the primary antibodies against ICAM-1, VCAM-1, Stat1, MOMA-2 as described previously [59]. Antibodies against ICAM-1, VCAM-1 were purchased from Santa Cruz, MOMA2 was purchased from BioRad and was used at 1:100. Appropriate secondary antibody was purchased from Jackson ImmunoResearch. Immunofluorescence microscopy was performed with Zeiss LSM800 laser confocal microscope.

### Statistical analysis

Data were expressed as mean ± SEM. Statistical difference was analyzed with the Student’s *t*-test. A value of P<0.05 was considered statistically significant.

### Ethics statements

All animal experiments were performed under approval from the Institutional Animal Care and Use Committee of the China Pharmaceutical University.

## Supplementary Material

Supplementary Figure 1

## References

[1] Libby P, Ridker PM, Hansson GK. Progress and challenges in translating the biology of atherosclerosis. Nature. 2011; 473:317–25. 10.1038/nature10146 21593864

[2] Ross R. Atherosclerosis—an inflammatory disease. N Engl J Med. 1999; 340:115–26. 10.1056/NEJM199901143400207 9887164

[3] Niemann B, Rohrbach S, Miller MR, Newby DE, Fuster V, Kovacic JC. Oxidative stress and cardiovascular risk: obesity, diabetes, smoking, and pollution: part 3 of a 3-part series. J Am Coll Cardiol. 2017; 70:230–51. 10.1016/j.jacc.2017.05.043 28683970 PMC5568826

[4] Gimbrone MA Jr, García-Cardeña G. Vascular endothelium, hemodynamics, and the pathobiology of atherosclerosis. Cardiovasc Pathol. 2013; 22:9–15. 10.1016/j.carpath.2012.06.006 22818581 PMC4564111

[5] Vanhoutte PM, Shimokawa H, Feletou M, Tang EH. Endothelial dysfunction and vascular disease - a 30th anniversary update. Acta Physiol (Oxf). 2017; 219:22–96. 10.1111/apha.12646 26706498

[6] Williams JW, Huang LH, Randolph GJ. Cytokine circuits in cardiovascular disease. Immunity. 2019; 50:941–54. 10.1016/j.immuni.2019.03.007 30995508 PMC6924925

[7] Libby P. Inflammation in atherosclerosis. Nature. 2002; 420:868–74. 10.1038/nature01323 12490960

[8] Herzig S, Shaw RJ. AMPK: guardian of metabolism and mitochondrial homeostasis. Nat Rev Mol Cell Biol. 2018; 19:121–35. 10.1038/nrm.2017.95 28974774 PMC5780224

[9] Yang Z, Kahn BB, Shi H, Xue BZ. Macrophage alpha1 AMP-activated protein kinase (alpha1AMPK) antagonizes fatty acid-induced inflammation through SIRT1. J Biol Chem. 2010; 285:19051–59. 10.1074/jbc.M110.123620 20421294 PMC2885183

[10] Speirs C, Williams JJ, Riches K, Salt IP, Palmer TM. Linking energy sensing to suppression of JAK-STAT signalling: a potential route for repurposing AMPK activators? Pharmacol Res. 2018; 128:88–100. 10.1016/j.phrs.2017.10.001 29037480

[11] Bess E, Fisslthaler B, Frömel T, Fleming I. Nitric oxide-induced activation of the AMP-activated protein kinase α2 subunit attenuates IκB kinase activity and inflammatory responses in endothelial cells. PLoS One. 2011; 6:e20848. 10.1371/journal.pone.0020848 21673972 PMC3108981

[12] Nerstedt A, Johansson A, Andersson CX, Cansby E, Smith U, Mahlapuu M. AMP-activated protein kinase inhibits IL-6-stimulated inflammatory response in human liver cells by suppressing phosphorylation of signal transducer and activator of transcription 3 (STAT3). Diabetologia. 2010; 53:2406–16. 10.1007/s00125-010-1856-z 20652679

[13] Dodington DW, Desai HR, Woo M. JAK/STAT - emerging players in metabolism. Trends Endocrinol Metab. 2018; 29:55–65. 10.1016/j.tem.2017.11.001 29191719

[14] Gharavi NM, Alva JA, Mouillesseaux KP, Lai C, Yeh M, Yeung W, Johnson J, Szeto WL, Hong L, Fishbein M, Wei L, Pfeffer LM, Berliner JA. Role of the jak/STAT pathway in the regulation of interleukin-8 transcription by oxidized phospholipids *in vitro* and in atherosclerosis *in vivo*. J Biol Chem. 2007; 282:31460–68. 10.1074/jbc.M704267200 17726017

[15] Agrawal S, Febbraio M, Podrez E, Cathcart MK, Stark GR, Chisolm GM. Signal transducer and activator of transcription 1 is required for optimal foam cell formation and atherosclerotic lesion development. Circulation. 2007; 115:2939–47. 10.1161/CIRCULATIONAHA.107.696922 17533179

[16] Yao QQ, Wang Y, Yang SM, Wang MZ. [Biotransformation of (+)- and (-)-clausenamide in rats]. Yao Xue Xue Bao. 2001; 36:224–28. 12580093

[17] Wang Y, Ruan W, Mi J, Xu J, Wang H, Cao Z, Saavedra JM, Zhang L, Lin H, Pang T. Balasubramide derivative 3C modulates microglia activation via CaMKKβ-dependent AMPK/PGC-1α pathway in neuroinflammatory conditions. Brain Behav Immun. 2018; 67:101–17. 10.1016/j.bbi.2017.08.006 28803158

[18] Chu SF, Zhang JT. Recent advances in the study of (-) clausenamide: chemistry, biological activities and mechanism of action. Acta Pharm Sin B. 2014; 4:417–23. 10.1016/j.apsb.2014.10.004 26579412 PMC4629111

[19] He C, Li H, Viollet B, Zou MH, Xie Z. AMPK suppresses vascular inflammation *in vivo* by inhibiting signal transducer and activator of transcription-1. Diabetes. 2015; 64:4285–97. 10.2337/db15-0107 25858560 PMC4657575

[20] He C, Medley SC, Hu T, Hinsdale ME, Lupu F, Virmani R, Olson LE. PDGFRβ signalling regulates local inflammation and synergizes with hypercholesterolaemia to promote atherosclerosis. Nat Commun. 2015; 6:7770. 10.1038/ncomms8770 26183159 PMC4507293

[21] Chiu JJ, Chien S. Effects of disturbed flow on vascular endothelium: pathophysiological basis and clinical perspectives. Physiol Rev. 2011; 91:327–87. 10.1152/physrev.00047.2009 21248169 PMC3844671

[22] Park JG, Ryu SY, Jung IH, Lee YH, Kang KJ, Lee MR, Lee MN, Sonn SK, Lee JH, Lee H, Oh GT, Moon K, Shim H. Evaluation of VCAM-1 antibodies as therapeutic agent for atherosclerosis in apolipoprotein E-deficient mice. Atherosclerosis. 2013; 226:356–63. 10.1016/j.atherosclerosis.2012.11.029 23245509

[23] Murase T, Mizuno T, Omachi T, Onizawa K, Komine Y, Kondo H, Hase T, Tokimitsu I. Dietary diacylglycerol suppresses high fat and high sucrose diet-induced body fat accumulation in C57BL/6J mice. J Lipid Res. 2001; 42:372–78. 11254749

[24] Taguchi H, Watanabe H, Onizawa K, Nagao T, Gotoh N, Yasukawa T, Tsushima R, Shimasaki H, Itakura H. Double-blind controlled study on the effects of dietary diacylglycerol on postprandial serum and chylomicron triacylglycerol responses in healthy humans. J Am Coll Nutr. 2000; 19:789–96. 10.1080/07315724.2000.10718079 11194533

[25] Cybulsky MI, Cheong C, Robbins CS. Macrophages and dendritic cells: partners in atherogenesis. Circ Res. 2016; 118:637–52. 10.1161/CIRCRESAHA.115.306542 26892963

[26] Chistiakov DA, Melnichenko AA, Myasoedova VA, Grechko AV, Orekhov AN. Mechanisms of foam cell formation in atherosclerosis. J Mol Med (Berl). 2017; 95:1153–65. 10.1007/s00109-017-1575-8 28785870

[27] Bentzon JF, Otsuka F, Virmani R, Falk E. Mechanisms of plaque formation and rupture. Circ Res. 2014; 114:1852–66. 10.1161/CIRCRESAHA.114.302721 24902970

[28] Ivashkiv LB. IFNγ: signalling, epigenetics and roles in immunity, metabolism, disease and cancer immunotherapy. Nat Rev Immunol. 2018; 18:545–58. 10.1038/s41577-018-0029-z 29921905 PMC6340644

[29] Yu XH, Zhang J, Zheng XL, Yang YH, Tang CK. Interferon-γ in foam cell formation and progression of atherosclerosis. Clin Chim Acta. 2015; 441:33–43. 10.1016/j.cca.2014.12.007 25512166

[30] Lim WS, Timmins JM, Seimon TA, Sadler A, Kolodgie FD, Virmani R, Tabas I. Signal transducer and activator of transcription-1 is critical for apoptosis in macrophages subjected to endoplasmic reticulum stress *in vitro* and in advanced atherosclerotic lesions *in vivo*. Circulation. 2008; 117:940–51. 10.1161/CIRCULATIONAHA.107.711275 18227389 PMC2276635

[31] Liu SY, Sanchez DJ, Aliyari R, Lu S, Cheng G. Systematic identification of type I and type II interferon-induced antiviral factors. Proc Natl Acad Sci USA. 2012; 109:4239–44. 10.1073/pnas.1114981109 22371602 PMC3306696

[32] Yusuf S, Hawken S, Ounpuu S, Dans T, Avezum A, Lanas F, McQueen M, Budaj A, Pais P, Varigos J, Lisheng L, and INTERHEART Study Investigators. Effect of potentially modifiable risk factors associated with myocardial infarction in 52 countries (the INTERHEART study): case-control study. Lancet. 2004; 364:937–52. 10.1016/S0140-6736(04)17018-9 15364185

[33] Cool B, Zinker B, Chiou W, Kifle L, Cao N, Perham M, Dickinson R, Adler A, Gagne G, Iyengar R, Zhao G, Marsh K, Kym P, et al. Identification and characterization of a small molecule AMPK activator that treats key components of type 2 diabetes and the metabolic syndrome. Cell Metab. 2006; 3:403–16. 10.1016/j.cmet.2006.05.005 16753576

[34] Foretz M, Carling D, Guichard C, Ferré P, Foufelle F. AMP-activated protein kinase inhibits the glucose-activated expression of fatty acid synthase gene in rat hepatocytes. J Biol Chem. 1998; 273:14767–71. 10.1074/jbc.273.24.14767 9614076

[35] Li Y, Xu S, Mihaylova MM, Zheng B, Hou X, Jiang B, Park O, Luo Z, Lefai E, Shyy JY, Gao B, Wierzbicki M, Verbeuren TJ, et al. AMPK phosphorylates and inhibits SREBP activity to attenuate hepatic steatosis and atherosclerosis in diet-induced insulin-resistant mice. Cell Metab. 2011; 13:376–88. 10.1016/j.cmet.2011.03.009 21459323 PMC3086578

[36] Ma A, Wang J, Yang L, An Y, Zhu H. AMPK activation enhances the anti-atherogenic effects of high density lipoproteins in apoE^-/-^ mice. J Lipid Res. 2017; 58:1536–47. 10.1194/jlr.M073270 28611100 PMC5538277

[37] Salt IP, Hardie DG. AMP-activated protein kinase: an ubiquitous signaling pathway with key roles in the cardiovascular system. Circ Res. 2017; 120:1825–41. 10.1161/CIRCRESAHA.117.309633 28546359 PMC5447810

[38] Assifi MM, Suchankova G, Constant S, Prentki M, Saha AK, Ruderman NB. AMP-activated protein kinase and coordination of hepatic fatty acid metabolism of starved/carbohydrate-refed rats. Am J Physiol Endocrinol Metab. 2005; 289:E794–800. 10.1152/ajpendo.00144.2005 15956049

[39] Sag D, Carling D, Stout RD, Suttles J. Adenosine 5'-monophosphate-activated protein kinase promotes macrophage polarization to an anti-inflammatory functional phenotype. J Immunol. 2008; 181:8633–41. 10.4049/jimmunol.181.12.8633 19050283 PMC2756051

[40] Fullerton MD, Ford RJ, McGregor CP, LeBlond ND, Snider SA, Stypa SA, Day EA, Lhoták Š, Schertzer JD, Austin RC, Kemp BE, Steinberg GR. Salicylate improves macrophage cholesterol homeostasis via activation of ampk. J Lipid Res. 2015; 56:1025–33. 10.1194/jlr.M058875 25773887 PMC4409279

[41] Cao Q, Cui X, Wu R, Zha L, Wang X, Parks JS, Yu L, Shi H, Xue B. Myeloid deletion of α1AMPK exacerbates atherosclerosis in LDL receptor knockout (LDLRKO) mice. Diabetes. 2016; 65:1565–76. 10.2337/db15-0917 26822081 PMC4878417

[42] Reiss AB, Patel CA, Rahman MM, Chan ES, Hasneen K, Montesinos MC, Trachman JD, Cronstein BN. Interferon-gamma impedes reverse cholesterol transport and promotes foam cell transformation in THP-1 human monocytes/macrophages. Med Sci Monit. 2004; 10:BR420–25. 15507847

[43] Wuttge DM, Zhou X, Sheikine Y, Wågsäter D, Stemme V, Hedin U, Stemme S, Hansson GK, Sirsjö A. CXCL16/SR-PSOX is an interferon-gamma-regulated chemokine and scavenger receptor expressed in atherosclerotic lesions. Arterioscler Thromb Vasc Biol. 2004; 24:750–5. 10.1161/01.ATV.0000124102.11472.36 14988089

[44] Voloshyna I, Littlefield MJ, Reiss AB. Atherosclerosis and interferon-γ: new insights and therapeutic targets. Trends Cardiovasc Med. 2014; 24:45–51. 10.1016/j.tcm.2013.06.003 23916809 PMC3844070

[45] Whitman SC, Ravisankar P, Elam H, Daugherty A. Exogenous interferon-gamma enhances atherosclerosis in apolipoprotein E-/- mice. Am J Pathol. 2000; 157:1819–24. 10.1016/s0002-9440(10)64820-1 11106554 PMC1885762

[46] Jin L, Waterman PM, Jonscher KR, Short CM, Reisdorph NA, Cambier JC. MPYS, a novel membrane tetraspanner, is associated with major histocompatibility complex class II and mediates transduction of apoptotic signals. Mol Cell Biol. 2008; 28:5014–26. 10.1128/MCB.00640-08 18559423 PMC2519703

[47] Ishikawa H, Barber GN. STING is an endoplasmic reticulum adaptor that facilitates innate immune signalling. Nature. 2008; 455:674–78. 10.1038/nature07317 18724357 PMC2804933

[48] Zhong B, Yang Y, Li S, Wang YY, Li Y, Diao F, Lei C, He X, Zhang L, Tien P, Shu HB. The adaptor protein MITA links virus-sensing receptors to IRF3 transcription factor activation. Immunity. 2008; 29:538–50. 10.1016/j.immuni.2008.09.003 18818105

[49] Sun W, Li Y, Chen L, Chen H, You F, Zhou X, Zhou Y, Zhai Z, Chen D, Jiang Z. ERIS, an endoplasmic reticulum IFN stimulator, activates innate immune signaling through dimerization. Proc Natl Acad Sci USA. 2009; 106:8653–58. 10.1073/pnas.0900850106 19433799 PMC2689030

[50] Liu Y, Jesus AA, Marrero B, Yang D, Ramsey SE, Sanchez GA, Tenbrock K, Wittkowski H, Jones OY, Kuehn HS, Lee CR, DiMattia MA, Cowen EW, et al. Activated STING in a vascular and pulmonary syndrome. N Engl J Med. 2014; 371:507–18. 10.1056/NEJMoa1312625 25029335 PMC4174543

[51] Warner JD, Irizarry-Caro RA, Bennion BG, Ai TL, Smith AM, Miner CA, Sakai T, Gonugunta VK, Wu J, Platt DJ, Yan N, Miner JJ. STING-associated vasculopathy develops independently of IRF3 in mice. J Exp Med. 2017; 214:3279–92. 10.1084/jem.20171351 28951494 PMC5679177

[52] Kato Y, Park J, Takamatsu H, Konaka H, Aoki W, Aburaya S, Ueda M, Nishide M, Koyama S, Hayama Y, Kinehara Y, Hirano T, Shima Y, et al. Apoptosis-derived membrane vesicles drive the cGAS-STING pathway and enhance type I IFN production in systemic lupus erythematosus. Ann Rheum Dis. 2018; 77:1507–15. 10.1136/annrheumdis-2018-212988 29945921 PMC6161667

[53] Mao Y, Luo W, Zhang L, Wu W, Yuan L, Xu H, Song J, Fujiwara K, Abe JI, LeMaire SA, Wang XL, Shen YH. STING-IRF3 Triggers Endothelial Inflammation in Response to Free Fatty Acid-Induced Mitochondrial Damage in Diet-Induced Obesity. Arterioscler Thromb Vasc Biol. 2017; 37:920–29. 10.1161/ATVBAHA.117.309017 28302626 PMC5408305

[54] Luo X, Li H, Ma L, Zhou J, Guo X, Woo SL, Pei Y, Knight LR, Deveau M, Chen Y, Qian X, Xiao X, Li Q, et al. Expression of STING is increased in liver tissues from patients with NAFLD and promotes macrophage-mediated hepatic inflammation and fibrosis in mice. Gastroenterology. 2018; 155:1971–84.e4. 10.1053/j.gastro.2018.09.010 30213555 PMC6279491

[55] Ramanjulu JM, Pesiridis GS, Yang J, Concha N, Singhaus R, Zhang SY, Tran JL, Moore P, Lehmann S, Eberl HC, Muelbaier M, Schneck JL, Clemens J, et al. Design of amidobenzimidazole STING receptor agonists with systemic activity. Nature. 2018; 564:439–43. 10.1038/s41586-018-0705-y 30405246

[56] Haag SM, Gulen MF, Reymond L, Gibelin A, Abrami L, Decout A, Heymann M, van der Goot FG, Turcatti G, Behrendt R, Ablasser A. Targeting STING with covalent small-molecule inhibitors. Nature. 2018; 559:269–73. 10.1038/s41586-018-0287-8 29973723

[57] Ni Y, Zhao L, Yu H, Ma X, Bao Y, Rajani C, Loo LW, Shvetsov YB, Yu H, Chen T, Zhang Y, Wang C, Hu C, et al. Circulating unsaturated fatty acids delineate the metabolic status of obese individuals. EBioMedicine. 2015; 2:1513–22. 10.1016/j.ebiom.2015.09.004 26629547 PMC4634820

[58] He C, Medley SC, Kim J, Sun C, Kwon HR, Sakashita H, Pincu Y, Yao L, Eppard D, Dai B, Berry WL, Griffin TM, Olson LE. STAT1 modulates tissue wasting or overgrowth downstream from PDGFRβ. Genes Dev. 2017; 31:1666–78. 10.1101/gad.300384.117 28924035 PMC5647937

[59] He C, Choi HC, Xie Z. Enhanced tyrosine nitration of prostacyclin synthase is associated with increased inflammation in atherosclerotic carotid arteries from type 2 diabetic patients. Am J Pathol. 2010; 176:2542–49. 10.2353/ajpath.2010.090783 20348234 PMC2861118

