# Supplementary material for: Balasubramide derivative 3C attenuates atherosclerosis in apolipoprotein E-deficient mice: role of AMPK-STAT1-STING signaling pathway
Source: Aging (Albany NY). 2021 Apr 26;13(8):12160–78. doi: 10.18632/aging.202929 (PMC8109080; doi:10.18632/aging.202929)
Supplement: Supplementary Figure 1 [file aging-13-202929-s001.pdf]

## SUPPLEMENTARY FIGURE

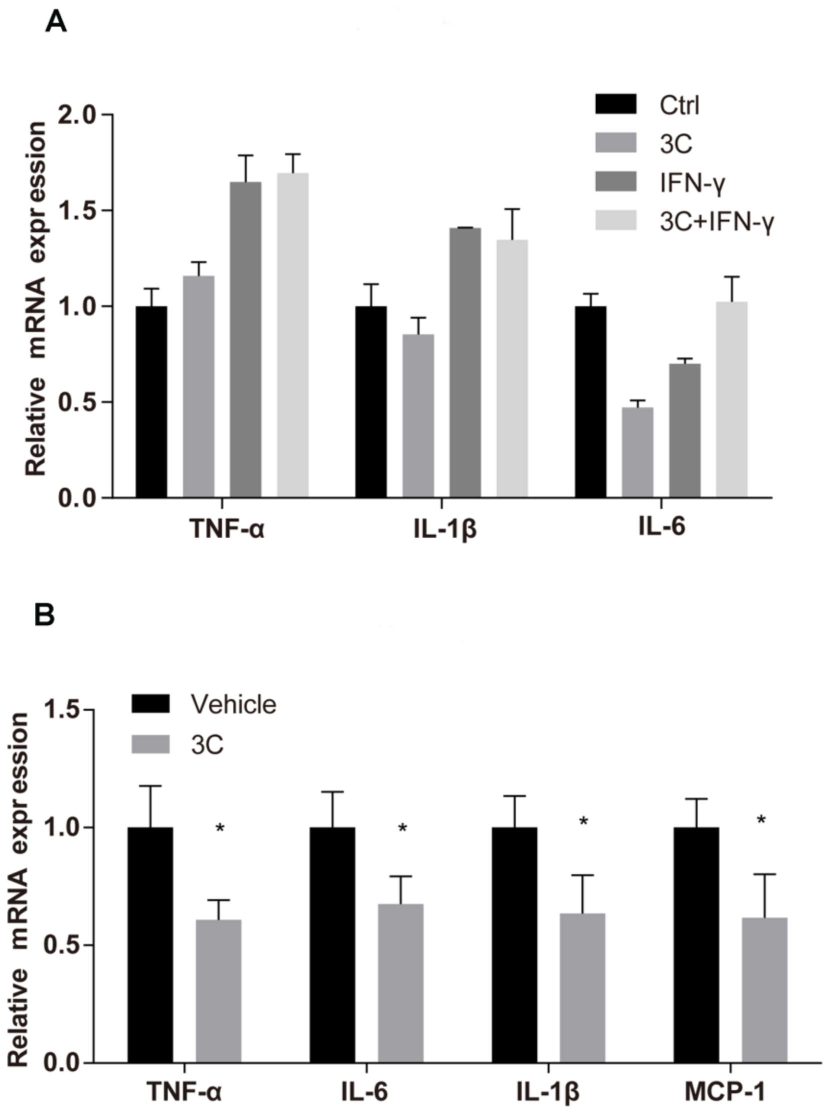


**Supplementary Figure 1.** (**A**) Relative gene expression of TNF-α, IL-β, IL-6 normalize to β-Actin. The experiments were performed in triplicate and repeated at least three times on different days. (**B**) Fold change of TNF-α, IL-β, IL-6 and MCP-1 mRNA expression level from aortas of different groups (n=4). Results are expressed as means ± SEM. *$P<0.05$, **$P<0.01$.
